# Microhomology‐Associated Androgen Receptor Deletion and Enrichment of AR Copy‐Number Variants in Androgen Insensitivity Syndrome

**DOI:** 10.1155/humu/3178183

**Published:** 2026-11-13

**Authors:** Juliana Moreira Marques, Raquel Martinez Ramos, Nathália Da Roz D′Alessandre, Gabriela Der Agopian Guardia, Barbara Leitão Braga, Ana Caroline de Freitas Afonso, Elisa França Chaves, Mariana Ferreira de Assis Funari, Mirian Yumie Nishi, Paula Fontes Asprino, Sorahia Domenice, Pedro Alexandre Favoretto Galante, Berenice Bilharinho Mendonca, Rafael Loch Batista

**Affiliations:** ^1^ Developmental Endocrinology Unit Hormone and Molecular Genetics Laboratory (LIM/42) Division of Endocrinology Department of Internal Medicine University of São Paulo School of Medicine São Paulo São Paulo Brazil usp.br; ^2^ Molecular Oncology Center Hospital Sírio-Libanês São Paulo São Paulo Brazil hospitalsiriolibanes.org.br; ^3^ Large-Scale Sequencing Laboratory (SELA) University of São Paulo School of Medicine São Paulo São Paulo Brazil usp.br; ^4^ Department of Endocrinology. Instituto do Câncer do Estado de São Paulo (ICESP) University of São Paulo School of Medicine São Paulo São Paulo Brazil usp.br

**Keywords:** 46XY DSD, androgen insensitivity syndrome, androgen receptor, copy number variation, differences of sex development, microhomology-dependent repair

## Abstract

**Background:**

Copy number variations (CNVs) in the androgen receptor (*AR*) gene are an underrecognized cause of androgen insensitivity syndrome (AIS). Defining their breakpoint architecture and mutational mechanisms may improve diagnosis and genotype–phenotype correlation.

**Objectives:**

To characterize a pathogenic *AR* deletion identified in a proband with complete androgen insensitivity syndrome (CAIS) and investigate its mechanism of formation, and to assess the frequency and genomic distribution of *AR* CNVs in AIS through a review of published cases and comparison with population data.

**Materials and Methods:**

Exome sequencing, MLPA, PCR, and Sanger sequencing delineated a hemizygous deletion spanning *AR* Exons 6–8 in a 46,XY individual. Breakpoints were mapped with Biostrings/GenomicRanges. We collated *AR* CNVs from the literature and curated databases for AIS (*n* = 991) and contrasted them with CNVs in the general population (gnomAD‐SV v4.1.0; *n* = 31,523). Odds ratios and enrichment *p* values were calculated.

**Results:**

Breakpoint sequencing revealed an 8‐bp microhomology in reverse‐complement orientation at the junction, consistent with microhomology‐associated *AR* deletion. Across 991 AIS cases, 48 carried *AR* CNVs, significantly enriched versus controls (OR = 5.08; *p* = 1.14 × 10^−17^). Exon 2 was the most frequently involved and strongly associated with CAIS.

**Discussion:**

To our knowledge, this is the first report describing breakpoint‐associated microhomology in a pathogenic *AR* deletion, suggesting a possible role for microhomology‐dependent repair. The enrichment of *AR* CNVs in AIS supports including CNV detection in the molecular diagnostic workflow.

**Conclusion:**

CNVs are a clinically relevant, underdetected class of *AR* defects in AIS. Integrating CNV detection and precise delineation into diagnostic workflows can enhance diagnostic yield and support genotype–phenotype assessment, whereas breakpoint characterization can provide insights into the mutational mechanisms underlying *AR* deletions.

## 1. Introduction

The androgen receptor (*AR*) gene, encoding a nuclear receptor, is essential for androgen signaling that regulates the physiological response to androgens [1]. The *AR* gene is located on chromosome Xq12, comprising eight exons, seven introns, and encodes a 920‐amino‐acid protein [2]. Structurally, the AR protein shares characteristics with other nuclear receptors, containing four distinct functional domains: the N‐terminal domain (NTD) (Residues 1–555), the DNA‐binding domain (DBD) (Residues 556–623), the hinge domain (Residues 624–665), and the ligand‐binding domain (LBD) (Residues 666–920) [1].

Pathogenic variants in the *AR* gene are the molecular basis of androgen insensitivity syndrome (AIS), a condition characterized by resistance to androgen signaling [3]. Clinically, AIS is classified into three phenotypic categories: complete androgen insensitivity syndrome (CAIS), partial androgen insensitivity syndrome (PAIS), and mild androgen insensitivity syndrome (MAIS) [4]. CAIS typically presents as an inguinal hernia in childhood or primary amenorrhea in adolescence in a 46,XY individual with fully developed but undescended testes, typical female external genitalia, normal breast development, and sparse or absent pubic and axillary hair, reflecting a complete inability to respond to androgens [5]. PAIS manifests as undervirilized external genitalia, whereas MAIS is often associated with infertility or persistent gynecomastia in males [6, 7].

Structural variations, particularly copy number variations (CNVs), play a significant role in AIS but are often underappreciated in clinical practice. CNVs are genomic alterations that involve duplications or deletions of DNA segments, usually ranging from 1 kilobase to several megabases [8]. These alterations can encompass entire genes or regulatory regions, affecting gene expression, dosage, and function. CNVs have been implicated in numerous genetic disorders, but their specific contribution to AIS remains incompletely understood [9].

The analysis of CNVs in the *AR* gene is particularly important, as the X‐linked nature of the gene means that a single‐copy deletion will necessarily lead to complete gene loss in 46,XY individuals, resulting in severe androgen resistance. Large deletions within the *AR* gene, particularly those affecting the LBD, have been associated with CAIS, whereas smaller deletions may result in PAIS or MAIS, depending on the extent of residual receptor function [10, 11]. Identifying CNVs and their causal mechanisms in *AR* can provide crucial insights into genotype–phenotype correlations, improving diagnostic accuracy, prognostic predictions, and personalized treatment strategies. Among the mechanisms that give rise to CNVs, microhomology‐mediated end joining (MMEJ) has emerged as a key DNA repair pathway responsible for nonrecurrent genomic deletions [12]. MMEJ is an error‐prone form of double‐strand break (DSB) repair that relies on short (5–25 bp) regions of sequence identity, providing homology to align and ligate DNA ends. Although deletions are its most common outcome, related microhomology‐dependent repair processes, particularly polymerase theta‐mediated end joining, can also generate templated insertions, tandem duplications, and complex rearrangements [13]. Unlike homologous recombination (HR), MMEJ does not require extensive sequence similarity and has been implicated in structural variants underlying various genetic disorders. The phenotypic consequences of deletions depend on gene dosage and allelic context: Heterozygous deletions of haploinsufficient autosomal genes and deletions of hemizygous X‐linked genes can both have direct phenotypic effects. In 46,XY individuals, deletion of the sole *AR* allele can therefore directly cause AIS. Identifying the sequence context at *AR* deletion breakpoints may provide insight into the mutational forces driving AIS and support the role of microhomology‐dependent repair in mediating clinically relevant structural variations.

In this study, we performed a molecular characterization of a large *AR* gene deletion identified in a patient with CAIS. Additionally, we compared the frequency of *AR* CNVs reported among individuals with AIS with the frequency of *AR*‐overlapping deletions and duplications among XY individuals in gnomAD‐SV. We analyzed data from the *AR* mutation database, which included 48 CNVs from 991 individuals with AIS, and compared it with the gnomAD structural variant database (Version SV v4.1.0), which contained 313 CNVs occurences in 31,523 XY individuals serving as controls. The odds ratio (OR) was calculated to determine whether CNVs in *AR* are significantly enriched in AIS. This analysis is aimed at assessing whether CNVs in the *AR* region occur more frequently in AIS than in the general population, thus providing further evidence of the pathogenic relevance of CNVs in AIS.

## 2. Methods

### 2.1. Whole Exome Sequencing (WES)

WES was performed using the SureSelect XT Human All Exon V6 Kit (Agilent). Libraries were sequenced on the Illumina HiSeq 2500 platform, generating paired‐end reads (2 × 300 bp). Reads were aligned to the hg19 human genome assembly using BWA‐MEM (RRID: SCR_010910). The aligned reads were sorted, and duplicate reads were flagged using the bamsort and bammark duplicates tools from biobambam2 (RRID: SCR_003308). Variant calling for single‐nucleotide variants (SNVs) and small insertions/deletions (indels) was performed using FreeBayes (RRID: SCR_010761), and annotation was carried out with ANNOVAR (RRID: SCR_012821).

### 2.2. *AR* Gene Sequencing

Genomic DNA was extracted from peripheral blood leukocytes using the proteinase K‐SDS salting‐out method. The entire *AR* gene (ENST00000374690) coding region, including the exon–intron boundaries of Exons 1–8, was amplified using previously published PCR primers [14–16]. PCR products were sequenced using the ABI Prism BigDye Terminator Cycle Sequencing Ready Reaction Kit (Life Technologies, Carlsbad, California) and analyzed on the ABI Prism Genetic Analyzer 3130XL (Life Technologies, Carlsbad, California).

### 2.3. Multiplex Ligation‐Dependent Probe Amplification (MLPA)

Structural variations in the *AR* gene, initially identified by PCR, were confirmed using the SALSA MLPA Reagent Kit (MRC‐Holland) with the SALSA MLPA Probemix P074 *AR*, following the manufacturer′s protocol.

### 2.4. Breakpoint and Recombination Analysis

Breakpoint analysis was performed to investigate the recombination mechanism underlying the *AR* gene deletion. Patient and reference *AR* genomic sequences (GRCh38/hg38) were compared using the UCSC Genome Browser and further analyzed in R (Version 4.2.2) with Bioconductor packages Biostrings (Version 2.66.0) and GenomicRanges (Version 1.50.0). Additional dependencies included IRanges (Version 2.32.0), XVector (Version 0.36.0), and rtracklayer (Version 1.56.1). Analyses were conducted under Bioconductor release 3.16. A 200‐bp region flanking each side of the predicted deletion breakpoint was extracted for local alignment. The pairwiseAlignment function from Biostrings was used to align the upstream and downstream sequences, enabling the identification of homologous and nonhomologous segments involved in the recombination event. The exact breakpoint coordinates were mapped using GenomicRanges, allowing accurate annotation of the recombination site. Detection of short homologous sequences near the breakpoint was performed to explore potential signatures of DNA repair mechanisms.

### 2.5. Literature Review

A comprehensive literature review was conducted in PubMed from inception to August 10, 2025, without language restrictions. The search included the following keywords: (“androgen insensitivity syndrome” OR “AIS” OR “46,XY disorder of sex development” OR “46,XY DSD” OR “testicular feminization syndrome” OR “XY sex reversal” OR “male pseudohermaphroditism”) AND (“androgen receptor” OR “*AR* gene” OR “NR3C4” OR “dihydrotestosterone receptor” OR “testosterone receptor”) AND (“copy number variation” OR “CNV” OR “gene dosage” OR “genomic deletion” OR “genomic duplication” OR “gene copy number” OR “structural variation” OR “chromosomal aberration” OR “genomic rearrangement” OR “large deletion” OR “large insertion” OR “large indel” OR “gross deletion”).

### 2.6. Database Review

To investigate previously reported CNVs in the *AR* gene, genetic databases were reviewed, including the Androgen Receptor Database (http://androgendb.mcgill.ca/), the Human Gene Mutation Database (HGMD) (http://www.hgmd.cf.ac.uk/), and ClinVar (https://www.ncbi.nlm.nih.gov/clinvar/). Additionally, a secondary search was performed in the Genome Aggregation Database (gnomAD) (https://gnomad.broadinstitute.org) to ensure comprehensive data coverage. To assess the pathogenicity of CNVs in *AR*, a set of bioinformatic prediction tools was employed, including PolyPhen2, SIFT, REVEL, MetaLR, MetaRNN, MetaSVM, Mutation Assessor, MutationTaster, FATHMM, FATHMM‐MKL, LRT, M‐CAP, and DAAN (https://franklin.genoox.com/clinical-db/home).

### 2.7. Gene Enrichment Analysis

To assess whether CNVs in the *AR* gene are significantly enriched in individuals with AIS, we performed a comparative gene burden analysis using two independent datasets. The AIS group comprised 991 individuals, among whom 48 carried CNVs in *AR*, as identified through curated mutation databases. For comparison, in the control dataset from gnomAD‐SV (Version 4.1.0), we identified 313 CNVs counts involving *AR* among 31,523 XY individuals from the general population. From this, there were 17 unique CNVs in total, the most frequent one with 68 alleles in the XY population. Most AR‐overlapping CNVs were deletions, and none of them occurred in Exon 11 in introns, one in the UTR region and one in the promoter. The remaining four CNVs were duplications, and two of them were aligned within highly polymorphic repeat regions. For this comparison, only gnomAD‐SV variants annotated as deletions or duplications were included. An OR was calculated to estimate the relative likelihood of *AR* CNVs occurring in AIS cases versus the general population. Fisher′s exact test was used to evaluate statistical significance, with a two‐tailed *p* value (*p* < 0.05) considered significant.

Genomic coordinates of the CNVs from the patient cohort were mapped to the human reference genome (GRCh38/hg38) and visualized using Python. A heatmap was generated to display the frequency of CNVs in the cohort affecting each exon/intron combination. In parallel, CNVs were plotted across the genomic coordinates of the *AR* locus with color coding based on phenotype, allowing positional correlation with functional domains of the gene and potential phenotype‐specific clustering. This analysis is aimed at quantifying the burden of *AR* structural variation in AIS and determining whether CNVs in this gene represent a nonrandom, disease‐associated event.

### 2.8. Ethics Statement

Written informed consent was obtained from the patient for genetic and laboratory studies. This study was approved by the local ethical committee (CAAE: 66367717.6.0000.0068).

### 2.9. Data Availability

The AR deletion reported here was submitted to ClinVar with submission ID SUB16462128. Individual‐level sequencing data are available from the corresponding author upon reasonable request.

## 3. Results

### 3.1. Clinical and Hormonal Findings

A 24‐year‐old individual assigned female at birth, with a history of primary amenorrhea, presented for evaluation after multiple unsuccessful attempts to induce menstruation with progestin therapy during adolescence. She also had a history of a right‐sided inguinal hernia in early childhood, which had been managed conservatively.

On physical examination, she was in good general condition (BMI 25 kg/m^2^), with typical female external genitalia, Tanner Stage M5 breast development, and markedly sparse pubic hair. No gonads were palpable in the inguinal region.

Laboratory testing revealed elevated total testosterone, increased luteinizing hormone (LH), and normal follicle‐stimulating hormone (FSH) levels (Table S1). Cytogenetic analysis confirmed a 46,XY karyotype. Pelvic magnetic resonance imaging demonstrated the absence of the uterus, cervix, and upper vagina, along with bilateral gonadal‐like structures in the iliac fossae.

### 3.2. Molecular Characterization of a Deletion in the *AR* Gene

WES identified a deletion of approximately 2008 base pairs on chromosome Xq12, encompassing Exons 6, 7, and 8 of the *AR* gene (Figure 1A). These exons encode the majority of the LBD of the AR. No additional pathogenic variants were detected in other genes associated with disorders of sex development (DSD).

**Figure 1:**
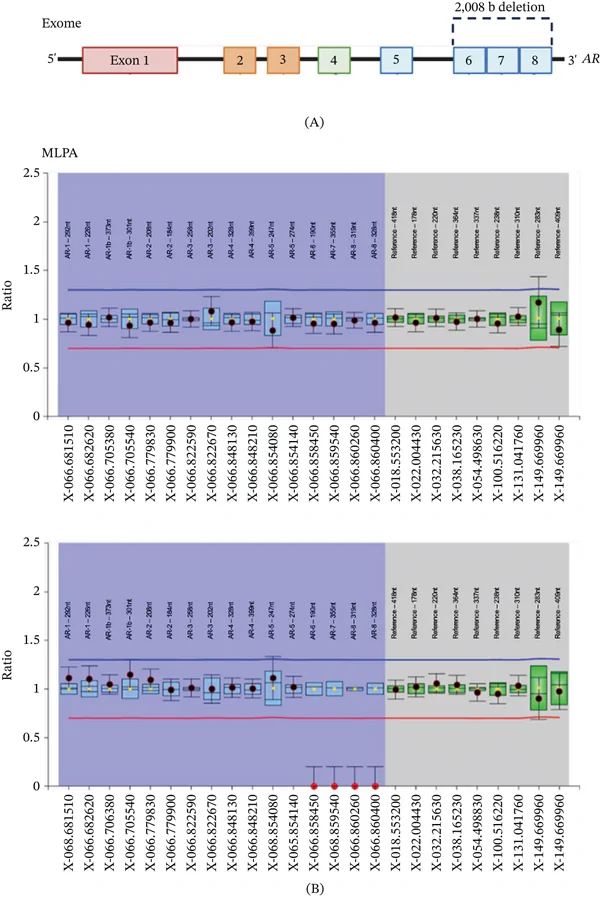
Identification and refinement of deletions in the *AR* gene using exome sequencing, MLPA (multiplex ligation‐dependent probe amplification), and Sanger sequencing. (A) Schematic representation of the *AR* gene exons (1–8), with a 2008‐bp deletion initially identified by exome sequencing, spanning Exons 6–8. (B) MLPA analysis identified a deletion encompassing Exons 6–8 of the *AR* gene. Probe signal intensities from the patient were compared with reference samples to assess copy number variation. The blue and red horizontal lines represent arbitrary thresholds set at approximately ±0.3 from the average probe value across reference samples. Blue and green boxes correspond to the 95% confidence intervals (CIs) for each probe in the reference group. Probes in the blue‐shaded zone target exonic regions of interest. Probes in the gray‐shaded zone serve as internal controls. Black dots represent patient probe ratios within the 95% CI; red dots fall outside this interval, suggesting altered copy number.

To validate and refine the identified structural variant, we employed MLPA, which revealed absent probe signal intensity for Exons 6–8, consistent with a hemizygous deletion (Figure 1B). PCR and Sanger sequencing further confirmed the deletion and defined its boundaries with higher resolution, revealing a 15,945‐bp ([GRCh38]chrX:67,718,479‐67,734,424) deletion that spanned the same exons. This discrepancy in size compared with the WES‐based call reflects the limited breakpoint resolution of short‐read exome data. The combined approach confirmed the loss of the *AR* LBD due to a large genomic deletion. This deletion was proven to be de novo in the proband due to its absence in the maternal X chromosomes, confirmed with MLPA and PCR.

To investigate the mechanism underlying the *AR* gene deletion, breakpoint analysis was performed using local sequence alignment of upstream and downstream flanking regions. The deleted segment was bordered by two 8‐bp microhomologous sequences located 3 bp upstream of the 5 ^′^ breakpoint and 32 bp downstream (in reverse‐complement orientation) of the 3 ^′^ breakpoint (Figure 2A). Together with the small insertions and retained nucleotides flanking the breakpoint junction, this configuration is compatible with a microhomology‐dependent end‐joining process, although the precise repair pathway cannot be determined from the available data. Local pairwise alignment confirmed the microhomology region and the absence of extensive homology required for HR (Figure 2B). Additionally, short insertions retained in the junction site (Figure 2C, lower case nucleotides), together with the offset position of the microhomology relative to the breakpoints, are consistent with template alignment, microhomology recognition, and resection‐dependent repair. Repetitive elements from the *Alu* and MIR subfamilies—both members of the SINE (short interspersed nuclear elements) family—were identified near both breakpoints (Figure 2D), although they were located at considerable distances from the deletion boundaries. Although no direct involvement was observed, the presence of these elements may have contributed to local genomic instability or structural susceptibility to breakage and microhomology repair.

**Figure 2:**
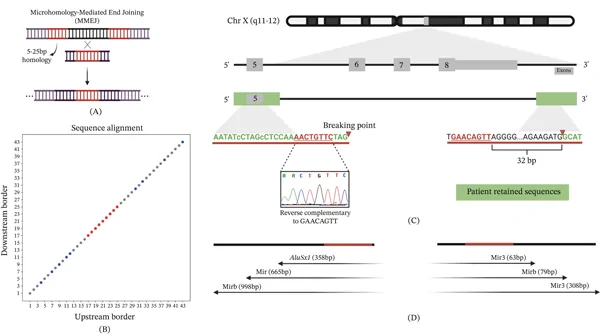
Microhomology at the breakpoints of the AR deletion. (A) Schematic of a microhomology‐dependent mechanism, such as microhomology‐mediated end joining (MMEJ), which utilizes short homologous sequences (5–25 bp) to mediate end joining at the break site, often resulting in deletions. (B) Dot plot alignment of upstream and downstream breakpoint borders reveals a region of microhomology (red), consistent with a microhomology‐dependent repair process. (C) Genomic context of the *AR* gene on chromosome X, showing a deletion spanning Exons 6–8. A microhomology of 8 bp (GAACAGTT) was identified 3 bp upstream of the 5 ^′^ breakpoint and 32 bp downstream (within the deleted region) on the 3 ^′^ side in reverse complement orientation, consistent with a microhomology‐mediated mechanism. (D) *Alu* and MIR elements are present near the breakpoints but distant from the junction, suggesting an indirect role in promoting genomic instability and susceptibility to microhomology‐dependent repair.

### 3.3. Enrichment and Genomic Distribution of *AR* CNVs in AIS

To assess the pathogenic relevance of *AR* CNVs, we compared the frequency of structural variants in 991 individuals with AIS (48 CNVs) to that in 31,523 XY individuals from the gnomAD database (313 CNVs). The analysis revealed a significant enrichment of CNVs in AIS cases (OR = 5.08; *p* = 1.14 × 10^−17^), reinforcing the contribution of large deletions and duplications to the molecular etiology of AIS. A total of 48 CNVs affecting the *AR* gene were compiled and mapped by genomic region and clinical phenotype (Figure 3A,B, Table 1). The most frequently affected region was Exon 2, with 12 independent cases, followed by Exons 1 and 1–8 deletions. Heatmap analysis revealed a nonrandom distribution of CNVs, with clustering in exons encoding the DNA and LBDs (Figure 3A). When stratified by phenotype, 84% of the CNVs were associated with CAIS, 14% with PAIS, and 2% with MAIS. Notably, most deletions associated with CAIS involved Exon 2 or the entire 3 ^′^ portion of the gene, whereas PAIS‐linked CNVs were more frequently located in Intron 2 or partially spanned Exons 5–7, suggesting a possible genotype–phenotype correlation influenced by CNV extent and location.

**Figure 3:**
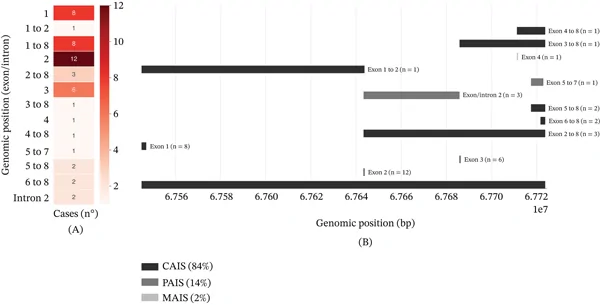
Genomic distribution and phenotypic correlation of CNVs in the *AR* gene. (A) Heatmap showing the number of reported CNV cases affecting specific regions of the *AR* gene. The highest concentration of CNVs was observed at Exon 2 (*n* = 12), followed by Exons 1 (*n* = 8) and 1–8 deletions (*n* = 8), supporting a recurrent breakpoint hotspot. (B) Genomic locations of CNVs mapped along the *AR* gene, color‐coded by associated phenotype: CAIS (dark gray), PAIS (medium gray), and MAIS (light gray). Exon 2 deletions represent the most frequent event (*n* = 12), predominantly associated with the most severe phenotype, CAIS. Relative proportions of CAIS (84%), PAIS (14%), and MAIS (2%) are indicated.

**Table 1:** Copy number variations (CNVs) in the *AR* gene in individuals with androgen insensitivity syndrome (AIS).

Phenotype	Number of cases	Variant type	Affected region	Reference
CAIS	1	Deletion	Exons 1–8	32
CAIS	1	Deletion	Exons 1–8	33
CAIS	1	Deletion	Exons 1–8	26
CAIS	2	Deletion	Exons 1–8	34
CAIS	2	Deletion	Exon 2 (7028 bp)	35
CAIS	3	Deletion	Exons 1–8	36
CAIS	2	Deletion	Exons 2–8	37
CAIS	2	Deletion	Exon 2	38
CAIS	2	Deletion	Exon 3	36
PAIS	1	Deletion	Exon 3	26
CAIS	1	Deletion	Exons 4–8	39
CAIS	1	Deletion	Exons 3–8	39
MAIS	1	Deletion	Exon 4	25
CAIS	1	Deletion	Exons 1–2	40
CAIS	2	Deletion	Exons 5–8	41
PAIS	1	Deletion	Intron 2 (> 6 kb)	41
CAIS	1	Deletion	Exon 3	42
CAIS	2	Deletion	Exon 3	43
PAIS	2	Deletion	Intron 2 (6 kb)	44
CAIS	1	Duplication	Exon 2	43
CAIS	1	Deletion	Exon 2	38
PAIS	1	Deletion	Exons 5–7	23
CAIS	2	Deletion	Exon 2	45
CAIS	2	Deletion	Exon 2	45
CAIS	7	Indel	Exon 1 del287ins77	46
CAIS	1	Deletion	Exons 2–8	22
CAIS	1	Duplication	Exon 2	21
PAIS	1	Deletion	Exon 1	22
PAIS	1	Deletion	Exon 2	22
CAIS	1	Deletion	Exons 6–8	Present study

To further explore genotype–phenotype correlations, we compiled reported CNVs in the *AR* gene from the literature and databases and mapped their distribution across the gene structure. As shown in Figure 3B, deletions affecting Exon 2 were the most frequently reported (*n* = 12), followed by deletions encompassing the entire gene or large segments (e.g., Exons 1–8 or 2–8, each with *n* = 8). Notably, Exon 2 and Intron 2 emerging as high‐density zones for genomic rearrangements.

## 4. Discussion

Identifying the molecular etiology of 46,XY DSD is essential for personalized care [17, 18]. In CAIS, early diagnosis facilitates timely decisions regarding gonadal management and gender‐affirming care [19]. In PAIS, the underlying variant may influence surgical planning, fertility counseling, and long‐term follow‐up [6, 20]. Integrating molecular and clinical data is crucial to improving outcomes in AIS.

This study contributes to the growing recognition that CNVs in the *AR* gene are a significant and often underrecognized cause of AIS. We describe a patient with a 46,XY karyotype, classical CAIS phenotype, and a large deletion encompassing Exons 6–8 of *AR*, corresponding to the LBD. The diagnosis was confirmed by orthogonal methodologies: WES, MLPA, PCR, and Sanger sequencing, highlighting the need for CNV‐sensitive methods when routine sequencing fails to detect pathogenic variants.

Recently, a novel duplication involving Exon 2 of the AR was reported in a patient with CAIS, which was missed by conventional sequencing but successfully detected through a WES pipeline with integrated CNV Calling 21. Their findings underscore the need for routine implementation of CNV‐sensitive approaches, including WES with dedicated CNV‐calling algorithms and MLPA, especially in patients with strong clinical suspicion but no identifiable SNVs. Our case reinforces the importance of detecting pathogenic AR deletions. Together with previously reported AR duplications, it supports the use of CNV‐sensitive methods capable of detecting both types of structural variation in the diagnostic evaluation of AIS.

By reviewing the literature, we identified 48 previously reported CNVs in the *AR* gene, most of which were associated with CAIS. These typically involved large deletions spanning multiple exons. Although less frequent, CNVs have also been reported in individuals with PAIS or even MAIS 22‐26, indicating that structural variants may underlie a broader spectrum of AIS phenotypes. Supporting the clinical relevance of these findings, a comparative frequency analysis demonstrated significant enrichment of *AR* CNVs in individuals with AIS compared with XY individuals from gnomAD‐SV (OR = 5.08; *p* = 1.14 × 10^−17^). Despite advances in sequencing technologies, structural variants remain underdiagnosed in AIS. Our findings emphasize the importance of integrating CNV analysis—through MLPA, WES‐based algorithms, or genome‐wide arrays—into routine diagnostic workflows, particularly in unsolved cases with high clinical suspicion.

Importantly, this is the first report to characterize breakpoint microhomology in a pathogenic AR deletion, a feature suggestive of microhomology‐dependent repair processes. Genomic deletions result from different DNA repair pathways activated in response to DSBs [21]. Whereas HR uses long identical DNA templates for accurate repair, nonhomologous end joining (NHEJ) and MMEJ operate without a template and are more error‐prone [22]. MMEJ relies on short microhomologous sequences (5–25 bp) to align DNA ends before ligation, frequently resulting in the loss of intervening DNA [13]. It has been increasingly recognized as a source of nonrecurrent deletions [23, 24]. It is particularly relevant in X‐linked genes, where single‐copy loss is sufficient to disrupt function in 46,XY individuals.

In this CAIS individual, genomic sequence alignment involving the flanking region of the deletion event revealed an 8‐bp microhomology at the junction, with no evidence of long direct repeats or extended homologous sequences. This pattern is compatible with a microhomology‐dependent end‐joining mechanism. Classical models of MMEJ involve annealing of short direct repeats in the same orientation on opposite 3 ^′^ overhangs, leading to deletion of the intervening sequence with retention of a single copy of the repeat. In our case, however, the 8‐bp motif is present in reverse‐complement orientation on the same strand, and therefore does not strictly fit the canonical MMEJ configuration. As a result, although MMEJ remains a plausible contributor, the available data do not allow us to distinguish unambiguously between canonical MMEJ, other microhomology‐dependent repair pathways, or more complex rearrangements (for example, a deletion coupled with local inversion). We therefore interpret this event as compatible with microhomology‐dependent repair rather than attributing it definitively to classical MMEJ.

Although MMEJ‐mediated deletions have not been previously reported in the *AR* gene, mobile element insertions have been reported in the AR 5 ^′^UTR region in individuals with PAIS [15, 25]. These insertions exhibit classical hallmarks of retrotransposition, such as target site duplications and poly(A) tails, and were associated with significant impairment of *AR* transcription. Although mechanistically distinct from MMEJ, these findings reinforce that noncanonical mutational mechanisms may contribute to monogenic disorders and highlight the vulnerability of the *AR* gene to structural rearrangements.

In conclusion, this study expands the mutational spectrum of the *AR* gene by characterizing a pathogenic deletion with a clearly defined 8‐bp microhomology at the breakpoint, compatible with microhomology‐dependent repair. Precise breakpoint mapping offers mechanistic insight and enhances genomic variant classification. These findings support the incorporation of genomic structural variant detection and, when deletions are identified, systematic breakpoint analysis into clinical diagnostics, advancing precision medicine in 46,XY DSD.

## Author Contributions

R.L.B., J.M.M., B.B.M., and P.A.F.G. conceived the study and designed the research. J.M.M., R.M.R., B.L.B., P.F.A., A.C.F.A., S.D., M.F.A.F., and M.Y.N. performed clinical phenotyping and laboratory experiments and quality control. P.A.F.G., G.D.A.G., E.F.C., and N.D.R.D. performed bioinformatic analysis. J.M.M. and R.L.B. prepared figures and visualizations. J.M.M., R.M.R., N.D.R.D., and R.L.B. drafted the manuscript. All authors contributed to data interpretation, critically revised the manuscript for important intellectual content, and approved the submitted and final versions.

## Funding

This study was supported by the Fundação de Amparo à Pesquisa do Estado de São Paulo (10.13039/501100001807; 2018/15579–8, 2019/26780‐9, 2024/23694‐2); (AACR) 2023 MONARCA (Maximizing Opportunity for New Advancements in Research in Cancer) Grant for Latin America (23‐15‐01‐BATI); Conselho Nacional de Desenvolvimento Científico e Tecnológico (10.13039/501100003593; 444825/2024‐0); and Hospital Sirio Libanes, Young Scientist program.

## Disclosure

A preprint has previously been published [26].

## Conflicts of Interest

The authors declare no conflicts of interest.

## Supporting information


**Supporting Information** Additional supporting information can be found online in the Supporting Information section. Table S1: Hormonal profile of the patient during diagnostic evaluation, including gonadotropins, sex steroids, and sex hormone‐binding globulin (SHBG).

## Data Availability

The datasets analyzed during the current study are publicly available on HGMD (https://www.hgmd.cf.ac.uk/), the androgen receptor mutations database (https://androgendb.mcgill.ca/), and the Genome Aggregation Database (gnomAD, https://gnomad.broadinstitute.org).
