# Supplementary material for: Microhomology‐Associated Androgen Receptor Deletion and Enrichment of AR Copy‐Number Variants in Androgen Insensitivity Syndrome
Source: Hum Mutat. 2026 Nov 13;2026:3178183. doi: 10.1155/humu/3178183 (PMC13768805; doi:10.1155/humu/3178183)
Supplement: Supplementary file 1 [file HUMU-2026-3178183-s001.docx]

**Suplemmentary Table 1.  Hormonal profile of the patient during diagnostic evaluation**

| **Lab exam** | **Exam 1** | **Exam 2** | **Exam 3** |
| --- | --- | --- | --- |
| FSH (4.5-21 mIU/mL) | 5.66 | - | 6 |
| LH (1-12 mIU/mL) | 26.52 | - | 23 |
| SHBG (20-95 nmol/L) | - | - | 62 |
| Androstenediona (<0.5 ng/mL) | - | - | 1.06 |
| Total Testosterone (12-60ng/dL) | 704 | 692 | 639 |
| Estradiol (20-157 pg/mL) | 46 | - | 55 |

Abbreviations: FSH – *Follicle-Stimulating Hormone*; LH – *Luteinizing Hormone*; SHBG – *Sex Hormone-Binding Globulin*;
